# Adult day services: a potential antidote to social isolation and loneliness in marginalized older adults

**DOI:** 10.3389/fpubh.2024.1427425

**Published:** 2024-09-06

**Authors:** Tina Sadarangani, Moroni Fernandez Cajavilca, Xiang Qi, William Zagorski

**Affiliations:** ^1^College of Nursing, New York University, New York, NY, United States; ^2^American Senior Care Centers Inc., Nashville, TN, United States

**Keywords:** adult day services, loneliness, long-term care, social isolation, older adults

## Abstract

Loneliness and social isolation affect more than 1 in 4 community-dwelling older adults in the United States, who may also require long-term care support. Despite being seen as a solution to the long-term care crisis, most older adults prefer to age in place rather than using skilled nursing facilities. However, in-home care is unsustainable due to a shortage of direct care workers and may exacerbate social isolation by confining older adults to their homes. Adult Day Services (ADS) addresses both issues. ADS provides care to adults with physical, functional, and or cognitive limitations in non-residential, congregate, community-based settings. ADS also provides daily cognitive and physical stimulation, often with medical support, in a social and supported environment, centered around the needs and preferences of participants. Before the COVID-19 pandemic, nearly 5,000 ADS centers were widely available. However, with limited public support, the ADS industry has struggled as demand by the growing number of older adults and families need health and social support. The ADS industry must be recognized for its unique ability to buffer social isolation and loneliness in chronically ill older adults while serving as an effective platform for chronic disease management. This perspective piece highlights the critical role of ADS centers in reducing loneliness and social isolation and promoting healthy equity. We also explore the benefits of ADS, the financial, policy, and societal barriers to utilizing ADS, and the potential solutions to ensure its sustainability and growth.

## Introduction

In the United States, 24% of adults over 60 experience social isolation, and 43% report feeling lonely (1, 2). Social isolation is the objective state of infrequent social contact with others, while loneliness is a subjective feeling of isolation (3). Older adults are at uniquely high risk of social isolation due to several factors that limit their ability to leave their homes and/or meaningfully and productively engage with others. These include living alone, having small social networks, lacking reliable transportation, experiencing a significant life event such as the loss of a spouse, and having mobility or sensory impairments that limit social engagement (3). The impacts of loneliness and social isolation on health are believed to be worse than smoking cigarettes (4). For example, loneliness has been associated with a 26% increased risk of premature mortality (5). In addition, social isolation and loneliness are associated with an increased risk of cognitive decline, dementia, depression, and cardiovascular disease (3, 6, 7).

While the policy landscape increasingly supports home and community-based services, there is the risk that keeping older adults at home—with just a few hours a week of support from a visiting nurse or home health aide—may exacerbate social isolation and loneliness. Home care confines older adults to their homes with limited opportunities for social interaction (8). For instance, a qualitative study found that home health nurses were unable to combat social isolation and loneliness among older adults as these phenomena were not accepted as a need for nursing care (9). Moreover, the direct care workforce shortage has made it increasingly difficult for families to access reliable and affordable in-home care services (10).

ADS centers allow older adults to remain in their community and simultaneously buffer loneliness and social isolation. ADS centers are non-residential, community-based, long-term care sites that provide health and social services in a congregate environment for a significant portion of the day (~8 h). ADS essentially enable older adults with physical and functional limitations to stay in their communities and receive the care they need, while also engaging with others and getting the care and supervision they need (11).

The purpose of this perspective piece is to highlight the critical role of ADS centers in reducing loneliness and social isolation and promoting healthy equity. We argue that ADS centers must be recognized and supported for their unique ability to buffer loneliness and social isolation in older adults. This perspective will also explore the benefits of ADS, the financial, policy, and societal barriers to the utilization of the centers, and the potential solutions to ensure its sustainability and growth.

## Adult day services

ADS centers provide community-based, person-centered care that emphasizes social connection and engagement, helping to combat the negative effects of isolation on older adults’ health and well-being. There are approximately 4,130 active ADS centers in the United States with about 251,100 users enrolled (12, 13). ADS users are medically complex, with a significant proportion living with dementia, diabetes, depression, or heart disease (11, 14). Furthermore, ADS users are more racially and ethnically diverse (55%) when compared to users of other long-term care services (15).

ADS centers offer a comprehensive range of services and activities that extend far beyond recreational activities and provide multifaceted support to meet the needs of diverse older adults and their caregivers (11). Cognitive stimulation is a key component of ADS; program directors design unique activities geared toward improving cognition while supporting peer-to-peer interactions (16). Daily exercise classes support physical health and mobility in a group environment (16). ADS centers bolster nutrition by offering balanced meals and snacks that cater to client’s dietary needs; dining areas support conversation and allow for communal celebrations that take place around meals (11). Regular health monitoring, including assessments and tracking of chronic conditions by trained healthcare professionals, including registered nurses, ensures that any potential issues are identified and addressed promptly (17). Moreover, ADS centers provide much-needed respite for family caregivers, allowing them to attend to their own needs and responsibilities while knowing their loved ones are in a safe and supportive environment (18).

## Addressing loneliness and health issues through ADS

Research has consistently demonstrated that ADS participation is associated with health-, social-, psychological-, and behavioral-related benefits for both care recipients and their caregivers, particularly in addressing social isolation, loneliness, and health-related issues among older adults. By providing a supportive and engaging environment, ADS participation can promote social connectedness and reduce feelings of loneliness (19, 20). A qualitative study by Dabelko-Schoeny and King (19) found that ADS participants reported increased opportunities for socialization, companionship, and a sense of belonging, which contributed to reduced feelings of loneliness and improved overall well-being. Similarly, a study by Iecovich and Biderman (20) found that ADS attendance was associated with a significant reduction in loneliness scores among older adults. A randomized controlled trial by Gitlin et al. (21) found that ADS Plus Program involved a staff social worker who provided care management and support to family caregivers will significantly reduce depressive symptoms and improve well-being among caregivers of impaired older adults enrolled in ADS. Similarly, a study by Schmitt et al. (22) found that ADS attendance was associated with a significant reduction in depressive symptoms and improved functional status among older adults.

Research also shows that ADS are the most racially diverse sector of long-term care, and centers benefit diverse older adults who are at disproportionately high risk of social isolation and loneliness because of factors such as non-English language preference and small social networks (3). ADS centers tend to be microcosms of the neighborhoods in which they are located; hence, centers within certain ethnic enclaves may cater to specific immigrant groups. Our previous study highlights that ADS centers successfully incorporate elements of older immigrants’ ethnic backgrounds and language into activities and programs that facilitate social connectedness, improve physical health and function, and preserve independence (23). The familiar environment, shared language, and cultural experiences provided by ADS centers offer a sense of belonging and support for older immigrants. Additionally, bilingual and bicultural staff, especially nurses, play a crucial role in promoting health literacy and transforming health care directives into culturally sensitive interventions (23).

## Discussion

### Future research directions

Future research on ADS and its impact on loneliness and social isolation should focus on several key areas. Firstly, there is a critical need for longitudinal studies that quantify the long-term effects of ADS participation on loneliness and social isolation measures specifically, using validated tools such as the UCLA Loneliness Scale or the Lubben Social Network Scale. These studies would provide robust evidence of the sustained benefits of ADS participation. Concurrently, researchers should investigate which specific components of ADS programs, such as group activities, one-on-one interactions, or cultural programs, are most effective in reducing social isolation and fostering meaningful social connections. This granular understanding would enable the development of more targeted and effective interventions. As technology continues to play an increasingly important role in healthcare delivery, examining the potential of hybrid ADS models that combine in-person and virtual participation is crucial. Such models could extend the reach and impact of ADS, particularly for older adults with mobility limitations or those in rural areas. Building on our previous findings (23), future studies should also explore the role of ADS in supporting social connections for older adults from diverse cultural backgrounds, ensuring that interventions are culturally appropriate and effective across different populations. Finally, conducting mixed-methods studies to understand the mechanisms by which ADS participation reduces loneliness and improves overall well-being, as suggested by the findings of Dabelko-Schoeny and King (19), would provide valuable insights for program development and implementation. By pursuing these research directions, we can strengthen the evidence base for ADS as an effective intervention for social isolation and loneliness, ultimately improving the quality of life for older adults.

### Funding and access to ADS

Despite the clear benefits of ADS, the COVID-19 pandemic threatened their viability. The pandemic negatively impacted the ADS industry, forcing many centers to close their doors and leaving vulnerable older adults without essential support and services. The forced closure of ADS centers in 2020 abruptly ended in-person services, exacerbating social isolation, caregiver burden, and accelerated cognitive and functional decline (24). The effects of these closures persist, with limited access to ADS centers due to staff loss and decreased government reimbursements (24). According to a survey conducted by the National Adult Day Services Association (NADSA), nearly 50% of ADS centers remained closed as of August 2020, with many facing financial hardship and uncertain futures (25). ADS centers will need to play an increasingly important role in the long-term care continuum amidst a burgeoning aging population, increasing rates of loneliness, and a shortage of direct care workers to provide in-home care. To prevent further closures and ensure the continued availability of these vital community-based resources, we call for immediate action to support and resource ADS centers.

Despite the clear health and social benefits of ADS centers, these centers remain underutilized and face significant financial and policy challenges. There are several reasons for this disconnect. First, there is a lack of public awareness about ADS and the vital role these centers play in supporting older adults and their caregivers. Many people are unfamiliar with the range of services offered by ADS and may incorrectly view them as merely providing recreational activities rather than comprehensive health and social support. Without a clear understanding of the benefits, families may be hesitant to enroll their loved ones. Second, access to ADS is limited by inadequate funding and reimbursement rates. Medicaid is a primary payer for ADS, but reimbursement rates vary widely by state and are often insufficient to cover the full cost of high-quality, person-centered services (26). ADS is primarily funded through a mix of public and private sources, including Medicaid, Veterans Administration, charitable grants, and private pay. This leaves ADS centers struggling financially, unable to hire and retain skilled staff, and forced to limit their hours and enrollment. For older adults who do not qualify for Medicaid, the out-of-pocket costs of ADS can be prohibitive. Finally, ADS centers have historically been overlooked in long-term care policy discussions, which tend to focus on institutional settings like nursing homes or home-based care. Additionally, many older adults and their family caregivers are unaware of the availability of ADS or may not meet the eligibility criteria for public funding, further limiting access to these services. There is a need for policies that specifically support and invest in ADS as a vital component of the long-term care continuum. This includes higher Medicaid reimbursement rates, inclusion of ADS in state Medicaid waivers, and efforts to collect and track quality data.

Addressing the underutilization of ADS will require a multi-pronged approach. Advocates must work to raise public awareness of the critical role of ADS in supporting healthy aging, while also pushing for policies and funding models that improve access and financial stability for these essential centers. To expand access and ensure the sustainability of ADS, it is essential to advocate for increased public funding, including higher Medicaid reimbursement rates and the inclusion of ADS in state Medicaid waivers (27). There is an ongoing effort to ensure ADS is a mandated benefit in all Medicaid plans.

By shining a light on both the challenges and the immense potential of ADS, we can build the public and political will to invest in this vital community resource. Furthermore, the lack of large-scale, standardized data on the impact of ADS closures during the pandemic represents a major obstacle to improving the health equity of community-dwelling older adults. To address this issue, ADS centers must prioritize the collection of race and ethnicity data and link it to quality measures of access to equitable, age-friendly care.

### Public rebranding and awareness campaign

To garner support for ADS and highlight its crucial role in the long-term care continuum, a public rebranding and awareness campaign is necessary (28). This campaign should aim to educate the public, policymakers, and healthcare professionals about the dangerous health impacts of loneliness and social isolation on older adults, and the comprehensive services provided by ADS centers that mitigate poor outcomes.

We suggest beginning this rebranding by moving away from the term “adult day care” in public marketing and advertising efforts as it is infantilizing and does not reflect the comprehensive health and social services that are provided. Instead, we propose using the term “Adult Day Services,” which better captures the range of services offered and emphasizes these programs’ health and social benefits. The campaign should utilize a multi-pronged approach. Engaging with local and national media outlets to feature stories highlighting the benefits of ADS and the experiences of participants and caregivers can be an effective strategy. This can include news articles, op-eds, and interviews with ADS providers, participants, and advocates. Developing a strong social media presence is also essential to share information, stories, and resources related to ADS. Using hashtags such as #AdultDayServices, #ADS, #AdultDayHealthCare, #SupportADS, #ReduceSocialIsolation, and #CombatLoneliness can increase visibility and engage with a wider audience.

Sharing testimonials and success stories from ADS participants and caregivers is a powerful way to humanize the impact of these services. These stories should focus on how ADS has helped reduce social isolation and loneliness, improved social connections, and enhanced the overall quality of life for participants (28). These stories can be featured on websites, social media, and in promotional materials. Key messages should emphasize the potential of ADS to reduce social isolation and loneliness, improve health outcomes, and delay or prevent institutionalization. The campaign should also highlight the cost-effectiveness of ADS compared to other long-term care options and its role in supporting aging in place and improving the quality of life for older adults and caregivers (28).

By raising awareness and reshaping public perceptions through a targeted and comprehensive campaign that emphasizes the role of ADS in reducing social isolation and loneliness, we can generate increased demand for ADS and mobilize support for policies that prioritize funding and access to these essential services.

## Conclusion

ADS centers may be an antidote to social isolation and loneliness. The COVID-19 pandemic has exposed the vulnerabilities of the ADS industry and the devastating consequences of abrupt closures on the social isolation and loneliness of older adults and their caregivers, particularly those from diverse racial and ethnic backgrounds. To address these challenges, a multi-faceted approach is necessary. Future research must focus on quantifying the long-term effects of ADS participation and exploring innovative service models. Increased public funding, higher reimbursement rates, and inclusion of ADS in Medicaid plans are essential to improve access. A comprehensive public awareness campaign is needed to educate stakeholders about the vital role of ADS in mitigating the health impacts of loneliness and social isolation. By investing in ADS through these measures, we can ensure that these valuable community-based resources continue to provide comprehensive, culturally sensitive care to our nation’s aging population, ultimately promoting health equity and improving the quality of life for older adults and their caregivers.

In summary, this perspective aims to highlight the disconnect between the clear benefits of ADS in combating social isolation and loneliness, and the financial and policy barriers that limit access to these services. By clarifying the underlying reasons for underutilization and identifying potential solutions, we hope to spur action to support and expand ADS as a key component of healthy aging in community settings. The absence of robust data on the impact of ADS closures during the pandemic underscores the need for greater investment in research and quality tracking to guide policy decisions and public investments in these essential centers moving forward.

## Data Availability

The original contributions presented in the study are included in the article/supplementary material, further inquiries can be directed to the corresponding author.
